# Gut–Lung Axis in Focus: Deciphering the Impact of Gut Microbiota on Pulmonary Arterial Hypertension

**DOI:** 10.3390/jpm14010008

**Published:** 2023-12-20

**Authors:** Konrad Suswał, Michał Tomaszewski, Aleksandra Romaniuk, Paulina Świechowska-Starek, Wojciech Zygmunt, Agnieszka Styczeń, Małgorzata Romaniuk-Suswał

**Affiliations:** 1 Department of Pulmonology, Alergollogy and Oncology, Medical University of Lublin, 20-954 Lublin, Poland; wojciech.zygmunt@umlub.pl; 2 Department of Cardiology, Medical University of Lublin, 20-954 Lublin, Poland; styczen.agnieszka@gmail.com; 3 Cardiology Student Scientific Circle, Academy of Silesia, 40-555 Katowice, Poland; romaniuk.aleksandra98@wp.pl; 4 Primary Care Clinic, Non-Public Healthcare Facility “Medycyna 2001”, 20-470 Lublin, Poland; pswiechowska.kontakt@gmail.com; 5 Department of Psychiatry, Psychotheraphy and Early Intervention, Medical University of Lublin, 20-954 Lublin, Poland

**Keywords:** pulmonary arterial hypertension, gut microbiota, metabolites and biomarkers, intestinal dysbiosis, microbial composition analysis

## Abstract

Recent advancements in the understanding of pulmonary arterial hypertension (PAH) have highlighted the significant role of the gut microbiota (GM) in its pathogenesis. This comprehensive review delves into the intricate relationship between the GM and PAH, emphasizing the influence of gut microbial composition and the critical metabolites produced. We particularly focus on the dynamic interaction between the gut and lung, examining how microbial dysbiosis contributes to PAH development through inflammation, altered immune responses, and changes in the gut–lung axis. Noteworthy findings include variations in the ratios of key bacterial groups such as *Firmicutes* and *Bacteroidetes* in PAH and the pivotal roles of metabolites like trimethylamine N-oxide (TMAO), short-chain fatty acids (SCFAs), and serotonin in the disease’s progression. Additionally, the review elucidates potential diagnostic biomarkers and novel therapeutic approaches, including the use of probiotics and fecal microbiota transplantation, which leverage the gut microbiota for managing PAH. This review encapsulates the current state of research in this field, offering insights into the potential of gut microbiota modulation as a promising strategy in PAH diagnosing and treatment.

## 1. Introduction

The gut microbiota are a complex community of microorganisms inhabiting the intestines. Their composition is diverse and consists of bacteria, fungi, archaeons, and viruses. The GM are actively involved in digestion, vitamin production, protection against pathogens, and also regulation of the immune system [1,2]. In addition, the metabolites that are produced by the microbiome affect the function of the intestinal barrier [3]. The main microorganisms comprising the gut microbiota include *Firmicutes*, *Bacteroides*, *Actinobacteria*, *Proteobacteria,* and *Verrucomicrobia*—with *Bacteroides* and *Firmicutes* accounting for 90% [4,5]. It is estimated that 99% of intestinal bacteria belong to about 40 species [6]. The composition of the gut microbiota stabilizes between the first and second years of life, and its composition depends on diet, physical activity, and overall health status [7,8]. Disorders in the composition of the microbiota (dysbiosis) can cause the development of diseases such as asthma, diabetes, atherosclerosis, hypertension, heart failure, or PAH [9]. The progress in metagenomics, metabonomics, and microbiology has opened up new avenues for understanding the development of PAH. By leveraging these advancements, research on gut microbiota and PAH offers a fresh perspective on unraveling the mechanisms underlying the onset of PAH. In this review, we explore the existing literature and offer insights into the influence of gut microbiota on PAH, providing a comprehensive overview of the current state of knowledge in this field.

## 2. Changes in the Gut Microbiota Composition in PAH

There is no doubt that the human microbiome influences human health and the development of various diseases. The gut microbiota is of particular importance here because of its influence on the gut–lung axis and its involvement in the pathogenesis of respiratory diseases [10,11]. Hong Wo et al., in their rat experimental model, demonstrated reduced diversity of the gut microbiota in rats with PAH and the involvement of a calcium-sensitive receptor antagonist (NPS2143) in reversing these abnormalities. In this experiment, Wistar rats were divided into three groups (control, PAH, and PAH + NPS2143). Induction of PAH was performed by injection of monocrotaline (MCT). A significantly lower diversity of gut microbiota was found in rats with PAH compared to the control group, and recovery after treatment with NPS2143 was observed. This may indicate that NPS2143 treatment reduces lung damage in patients with PAH [12]. Callejo et al. administered to Wistar rats a VEGF receptor antagonist (SU5416) and generated conditions of hypoxia to induce PAH. Features of intestinal dysbiosis were demonstrated in rats with PAH. There was a threefold increase in the ratio of *Firmicutes* to *Bacteroidetes* compared to the control group. However, the relative number of *Firmicutes* families remained unchanged, while *Bacteroidetes* families were significantly less abundant in rats with PAH. Still, serum acetate concentrations in rats with PAH were lessened—indicating a reduced number of acetate-producing intestinal bacteria [13].

Similar conclusions were also reached by Semeda et al. In a group of rats with induced PAH (SU5416 + hypoxia), the *Firmicutes*/*Bacteroidetes* (F/B) ratio was higher than in the control group. Moreover, the number of *Bacteroidetes* and *Akkermansia*, which are involved in reducing inflammation, decreased. In contrast, the number of *Rosia* and *Prevotellacea*, which induce inflammation, increased [14]. Accordingly, a disturbed ratio of bacteria within the gut microbiota may be associated with suppression or induction of inflammation. In addition to an increased F/B ratio in rats with PAH, Sharma et al. showed an increased number of *Clostridiales* and *Aerococcaceae* bacteria, which were not present in control rats [15].

Luo L. et al., in their experimental model, divided rats into four groups (control, hypoxia, HySU, and MCT). In this study, the rats’ stools were analyzed every two weeks for dynamic changes in gut microbiota. After two weeks, the α-diversity of gut microbiota in HySU rats decreased compared to the control group. After four weeks, the differences in microbiota diversity in the two groups were obliterated. Interestingly, the variability of α-diversity in the HySu rats was similar to the group given MCT. This may indicate similarities of the gut microbiota in PAH type 1 and PAH type 3 [16].

There is growing evidence of changes in the gut microbiota in patients with PAH. Kim et al. compared the fecal microbiome of 18 patients with PAH type 1 and 13 controls and showed significant changes in the microbiome in patients with PAH 1. Accordingly, proline, ornithine, and arginine synthesis pathways were increased in the PAH 1 group. In addition, bacterial groups associated with trimethylamine N-oxide and purine metabolism were increased in PAH patients, while populations of bacteria responsible for butyrate (*Coprococcus*, *Lachnospiraceae*, *Butyvibrio Eubacterium*) and propionate (*Akkermansia*, *Bacteroides*) production were increased in the reference cohort. This indicates a unique composition of the microbiome specific to patients with PAH, which may open up new diagnostic and therapeutic approaches for the treatment of this disease. [17]

In contrast, Zhang et al. examined the oral and pharyngeal microbiome in patients with PAH. They took throat swabs from 118 PAH patients and 79 controls. As an outcome of this work, they found an increased presence of *Lautropia*, *Streptococcus,* and *Ralstonia* bacteria in PAH patients, compared to the controls, while *Haemophilus*, *Rothia*, *Granulicatella*, *Capnocytophage,* and *Sccharibacteria* predominated in healthy subjects. The presence of *Lautropia*, *Streptococcus,* and *Ralstonia* only in patients with PAH can be used as a potential biomarker in the diagnosis of PAH [18].

The study by Moutsoglou et al. examined 73 PAH patients, 15 family members of PAH patients, and 39 healthy individuals. They found an increased number of pro-inflammatory bacteria (*Bacteroides thetaiotaomicron*, *Parabacteroides distasonis,* and *Bacteroides vulgatus*) and a lower number of anti-inflammatory bacterial species (*Butyrivibrio* sp. and *Bifidobacterium angulatum*) in PAH patients compared to the control group [19].

## 3. Role of Gut Microbiome Metabolites in PAH

The gut microbiota consist of about 10–100 trillion bacteria. This gives them a huge potential to carry out chemical reactions and produce metabolites [20]. Some, such as short-chain fatty acids, tryptophan (5-HT), or trimethylamine N-oxide (TMAO), may contribute to the pathogenesis of PAH [21,22,23].

### 3.1. Trimethylamine N-Oxide (TMAO)

TMAO is an organic compound, the precursor of which is trimethylamine (TMA) produced by the intestinal microbiota. TMA is transported to the liver, where, with the participation of a flavin-containing monooxygenase, it is oxidized and converted to TMAO. A diet rich in fish, beef, or eggs contributes to an increase in the concentration of TMAO in the blood and urine [24]. In 2013, Wang et al. were the first to show that TMAO can be used to assess cardiovascular risk [25]. Subsequent studies have shown that TMAO produced by the gut microbiota is closely linked to atherosclerosis and myocardial infarction [26,27]. Elevated levels of TMAO also increase the risk of development of atrial fibrillation, hypertension and heart failure [28,29]. Plasma TMAO has been shown to play a role in the development of atherosclerosis as it induces foam cell production, increases leukocyte adhesion, has prothrombotic effects, and enhances oxidative stress, resulting in the release of proinflammatory cytokines [30,31].

Yang et al. showed that TMAO can be a useful biomarker also in the course of PAH. They divided a group of 124 PAH patients into two cohorts stratified by the 50th percentile of plasma TMAO levels. They found that patients with high TMAO levels had higher NT-proBNP, lower tricuspid annular plane systolic excursion (TAPSE) and lower ejection fraction, and were statistically classified in a higher WHO-FC group than patients with low TMAO levels. Moreover, TMAO levels were reduced by successful treatment and correlated with NT-proBNP reduction. Thus, TMAO may be useful as a potential biomarker of cardiac function and used in the treatment of PAH. This direction seems interesting and requires further research. In the same study, Yang et al. conducted an experiment on rats. One cohort received MCT, while the other received MCT and 3,3-dimethylbutanol (DMB), which is a TMAO inhibitor. It was observed that in the DMB group, there was a reduction in TMAO levels, along with a significant improvement in hemodynamic parameters and a reduction in right heart burden compared to the MCT group [22,32].

### 3.2. Short-Chain Fatty Acids

Short-chain fatty acids (SCFAs) are organic acids with strong hydrophilic properties. They are produced by the bacterial flora inhabiting the large intestine through the fermentation of indigestible carbohydrate residues [33]. The concentration of SCFAs in the proximal part of the colon is higher than in the distal part. This is due to the increased availability of water and hydrocarbonates in the proximal part of the colon [34]. Only 5% of SCFAs are excreted in the feces; the remaining 95% are absorbed by colon epithelial cells, from where they go directly to the plasma and from there, via the portal vein, to the liver, where they are further metabolized [35]. The main SCFAs found in the intestine are acetate, propionate, and butyrate, which occur in a ratio of 3:1:1. Of these, butyrate is the most important. It is produced by the condensation of two acetyl-CoA molecules by bacteria from the *Lachnospiraceae* and *Ruminococcaceae* families [36]. Butyrate is responsible for maintaining immune balance locally and systemically. Circulating in the plasma, butyrate stimulates the production of regulatory T cells and thus promotes the cellular immune response to the pathogen. In contrast, butyrate localized in the gut protects the epithelial barrier and limits the influx of pro-inflammatory cytokines [37,38].

In a study conducted on rodents, Pulgarin et al. showed that in a group of rats that were given butyrate, right ventricular pressure was reduced and hypertrophy was attenuated. In addition, butyrate had the effect of reducing the inflammatory process and inhibiting endothelial remodeling within the pulmonary vasculature [39].

In their rat model, Kim et al. showed that the number of SCFA-producing bacteria, including *Coprococcus*, *Butyrivibrio*, *Lachnospiraceae*, *Eubacterium,* and *Clostridia*, was reduced in a cohort with PAH. SCFA deficiency resulted in an increased intestinal permeability and increased oxidative metabolism, which further impaired intestinal barrier function [17]. Of note, increased intestinal permeability is associated with PAH [40].

### 3.3. Serotonin

Serotonin (5-HT) is a 5-hydroxyl derivative of tryptamine belonging to the biogenic amines. It is an important neurotransmitter in the central nervous system, while in the periphery, it acts as an autocoid. Serotonin is formed from L-tryptophan, which is converted to 5-hydroxytryptophan with the participation of tryptophan hydroxylase. The 5-hydroxytryptophan is then decarboxylated to produce serotonin. Serotonin is produced mainly (80%) in the enterochromaffin cells of the intestinal mucosa with the participation of *Candida*, *Streptococcus,* and *Escherichia* bacteria [41]. The remaining part is produced in the sutural nuclei of the brain and the pineal gland [42]. 5-HT is also produced by the pulmonary artery endothelium, where it interacts with pulmonary artery smooth muscle cells (PASMCs) in a paracrine manner. 5-HT enters PASMCs via serotonin transporter protein (SERT), then binds to the 5-HT1B receptor on the plasma membrane and activates contractile and proliferative signaling pathways. As a result, abnormal pulmonary artery constriction, impaired PASMC proliferation, and increased pulmonary artery vascular resistance occur, resulting in the development of PAH [43].

5HT1B receptors and serotonin synthesis inhibitors (TPH1) appear to play a key role in the pathophysiology of PAH. 5HT1B receptors inhibit the accumulation of cyclic AMP, resulting in an increase in [Ca^2+^] concentration and vasoconstriction. Increased vascular wall tension, inhibition of nitric oxide synthesis, and removal of vascular endothelium contribute to the enhancement of receptor activation [44,45]. These factors also influence the development of PAH, indicating that the 5HT1B receptor is involved in this condition [44,46].

Baranowska-Kuczko et al. studied the effects of the molecule LY393558 on 5HT1B receptors. The molecule LY393558 is an antagonist of the 5-HT1B, 5-HT1D, and 5-HT2A receptors and a serotonin transporter (SERT) inhibitor. LY393558 has been shown to have antagonistic effects on 5-HT1B receptors and, in combination with SERT inhibition, reduces vasoconstriction. In another study, LY393558 was shown to inhibit the proliferation of PASMCs and thus inhibit and retract the induced PAH in an experimental model [47].

Tryptophan hydroxylase 1 (TPH1) is an enzyme in which the function is to catalyze the hydroxylation reaction of the amino acid, tryptophan, converting it into 5-hydroxytryptophan (5-HTP). The 5-HTP is then converted to serotonin with the involvement of aromatic L-amino acid decarboxylase (AAAD). This process occurs mainly in the enterochromaffin cells of the gastrointestinal tract and neurons of the nervous system [48]. In their rat study, Aiello et al. showed that TPH1 inhibitors reduced serotonin levels in the lungs, intestines, and serum. This resulted in a significant reduction in pulmonary vascular pressure, a reduction in the thickness of the pulmonary vascular wall, and a reduced number of histamine and mast cells [49].

### 3.4. Lipopolysaccharides

Lipopolysaccharides (LPSs) are part of the cell membrane of Gram-negative bacteria, including those that are a component of GM (e.g., *E. coli* or *Salmonella*). They also act as endotoxins that induce inflammation within the body [50]. Lipopolysaccharides play a role in the pathogenesis of PAH. LPSs increase intestinal permeability, which causes metabolites formed with GM to enter the plasma. LPSs present in the plasma contribute to the release of pro-inflammatory cytokines, such as interleukin-1 (IL-1), interleukin-6 (IL-6), interleukin-8 (IL-8), and tumor necrosis factor-alpha (TNF-alpha) [51,52]. The consequence of cytokine release is inflammation within the pulmonary vasculature and consequent vasospasm and increased pulmonary pressure. In addition, inflammation leads to endothelial damage, which increases the likelihood of thrombosis [53]. 

**Figure 1:**
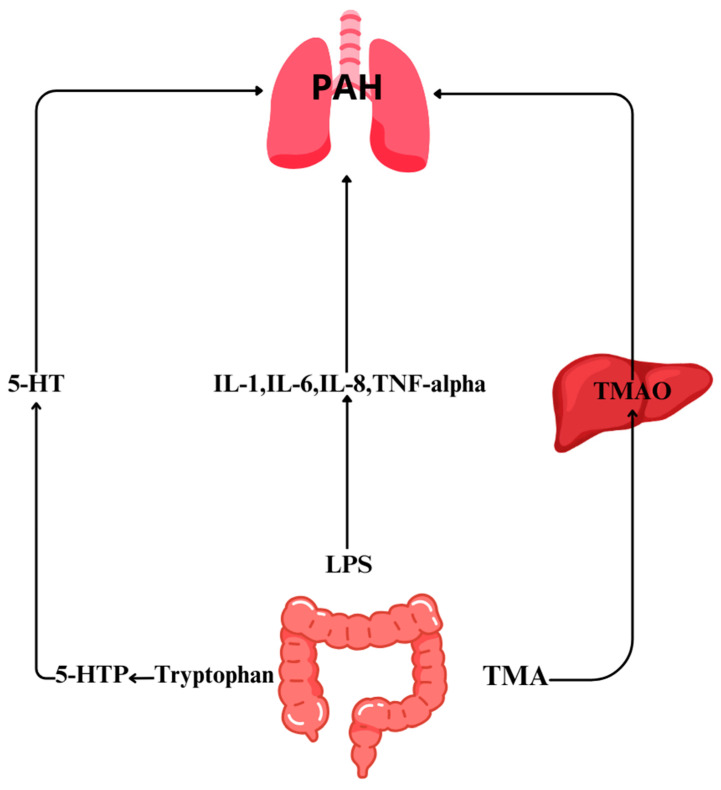
The synthesis and impact of trimethylamine N-oxide (TMAO), serotonin, and lipopolysaccharides (LPSs) on pulmonary arterial hypertension (PAH).

The synthesis and role of Gut Microbiome in PAH is highlighted in Figure 1.

## 4. Role of Other Diseases and Intestinal Dysbiosis in PAH

The development of PAH can result from the presence of other diseases, such as systemic lupus erythematosus, rheumatoid arthritis, portal hypertension, HIV infection, or schistosomiasis [15,54,55,56,57].

### 4.1. Systemic Lupus Erythematosus

Systemic Lupus Erythematosus is an autoimmune connective tissue disease that can affect various organs and systems in the human body. Luo et al., in their study, analyzed the intestinal flora of mice and patients with SLE. They showed that the composition of the microbiota between mice before the disease, and diseased mice and mice treated with immunosuppressive drugs differed significantly. They noted that increased numbers of lactic acid bacilli may be associated with progressive disease. In the same study, there were fewer differences in gut microbiota composition between healthy patients and patients with SLE. The intestinal microbiota in sick patients were less diverse, but it was noted that the number of Proteobacteria was significantly increased in patients with PAH [57].

### 4.2. Infection with Human Immunodeficiency Virus (HIV)

The HIV virus causes immunodeficiency in an infected person. It acts on the immune system, attacking mainly CD4+ lymphocytes. This leads to a weakening of the body’s defense functions and increases susceptibility to infection. The HIV virus is one of the risk factors for the development of PAH. It causes the release of cytokines that lead to inflammation and endothelial impairment within the pulmonary vasculature [58]. The role of HIV in PAH is the subject of much research. It is believed that HIV contributes to the impairment of the intestinal barrier, which causes the passage of intestinal bacteria and their metabolites into the systemic circulation [59]. Patients with HIV have been shown to have an altered composition of the gut microbiota compared to healthy individuals. Special attention should be attributed to significantly increased number of *P. aureginosa* and *C. albicans* bacteria in HIV patients compared to healthy individuals. In addition, HIV-positive subjects had elevated levels of fecal calprotectin, indicating chronic ongoing inflammation in the gut [60]. However, further studies are needed to investigate the relationship between changes in the composition of the gut microbiota during HIV and the development of PAH.

### 4.3. Schistosomiasis in PAH Development

Schistosomiasis is a parasitic disease caused by flukes of the Schistosoma species. Schistosomiasis infection occurs when cercariae (larvae of the parasite) penetrate human skin while in parasite-infested water. In the human body, the larvae develop and the adult forms end up in the blood vessels of organs such as the liver, lungs, and intestines, where they produce eggs [61]. In their study, Kumar et al. exposed mice to Schistosoma eggs. They showed that a single exposure to Schistosoma eggs causes the PAH phenotype, which spontaneously resolves after 1–2 weeks. In contrast, multiple exposures induced a persistent PAH phenotype accompanied by vascular fibrosis [62]. The association of Schistosoma Mansoni with modifications in the composition of the microbiota was also investigated by analyzing the feces of mice infected with *S. Mansoni* eggs. It turned out that compared to the control group, the microbiota of the diseased mice group contained a higher number of Bacteroides, Parabacteroides, Alistipes, and Helicobacter, while the number of Lactobacillus decreased [63,64]. The exact relationship between Schistosomiasis and the development of PAH is unknown and should be further investigated. 

## 5. Perspectives on Gut Microbiota in PAH Treatment

Studies conducted to date clearly indicate changes in the composition of the gut microbiota in patients with PAH, compared to healthy subjects. This opens up potential new possibilities for both diagnostic and therapeutic purposes. The composition of the microbiota is individual and depends on many factors, but it can also be dynamic and change depending on external and internal factors. The composition of the microbiota is influenced by gender, age, diet, lifestyle, diseases, medications taken, and environmental factors.

There is evidence of an association between a high-fat diet and changes in the composition of the intestinal microbiota by decreasing *Bacteroidetes* while increasing *Firmicutes* [65]. Other studies indicate that a diet rich in saturated fat may increase LPSs and TMAO and thus increase pulmonary vascular abnormalities and contribute to right ventricular hypertrophy [66,67]. In contrast, a diet rich in fiber is associated with increased production of SCFAs and especially acetate and butyrate, which reduced the development of PAH in rat models.

The Mediterranean diet is effective in reducing inflammation and increasing SCFA production in the gut. This reduces the risk of type II diabetes and cardiovascular diseases [68,69]. Diet is crucial in the primary prevention of cardiovascular diseases. However, the European Society of Cardiology and the European Respiratory Society have not specified specific dietary recommendations in the context of PAH. Still, in their study on mice, Vinke et al. showed that adding more leucine, protein, fish oil, and oligosaccharides to the feed reduced cardiac hypertrophy [70]. This may be an interesting direction, but one that requires more randomized trials. In patients with PAH, it seems reasonable to examine the nutritional status and, above all, to determine vitamins C and D and iron contents. A balanced diet with an adequate supply of macronutrients and micronutrients, especially with an adequate supply of iron, vitamins C and D, flavonoids, and fiber can have a positive impact on quality of life in patients with PAH [71,72,73].

Probiotics also play a role in reducing intestinal dysbiosis. Probiotics are live microorganisms that affect the balance of the intestinal microbiota and are beneficial to human health. The most important probiotics include strains of *Lactobacillus*, *Bifidobacterium*, *Saccharomyces* and *Streptococcus*. There are studies confirming the positive effects of probiotics on the human body by reducing levels of pro-inflammatory cytokines, lessening inflammation, and increasing SCFA production [74]. In their study on children, Wang et al. showed that probiotics administered to children with acute lung injury (ALI) decreased inflammation, improved lung function, and lowered pulmonary artery pressure [75].

Fecal microbiota transplantation (FMT) also seems to be an interesting direction for further research. With FMT, an appropriate and balanced composition of the intestinal microbiota is restored in the host organism. FMT has been successfully used to treat recurrent *Clostridium difficile* infections, antibiotic-resistant infections, and irritable bowel syndrome. Since the gut microbiota is significantly altered in PAH, it seems that FMT may be an intriguing therapeutic option for these patients. Currently, there is an interesting study underway in the United States in which FMT was performed in patients with PAH and then followed up for changes in microbiota composition and quality of life with PAH [76]. This study may shed new insights in the context of PAH treatment.

## 6. Conclusions

Studies suggest that the gut microbiota (GM) may play an important role in the pathogenesis of pulmonary arterial hypertension (PAH) by affecting the immune system, the gut–lung barrier, and metabolite production. A specific composition of the intestinal microbiota is observed in PAH patients compared to healthy individuals. Changes in the composition of the microbiota can lead to inflammation, increased permeability of the intestinal barrier, and immune dysfunction, which can contribute to the development and progression of the disease.

Studies are also pointing to potential biomarkers related to the gut microbiota, such as TMAO or SCFA levels, which may be useful in diagnosing and monitoring the disease. In addition, TPH1 inhibitors and 5-HT1B receptor antagonists are targets for research into potential therapies that may affect the development of PAH.

However, it should be noted that the topic of how the gut microbiota affect PAH requires further research and experimentation to better understand the mechanisms of action and potential therapeutic applications. The introduction of therapies based on the regulation of the gut microbiota may be a promising direction in the treatment of patients with PAH, but this still requires many more clinical studies.

## Data Availability

Not applicable.
